# Mechanical and Recyclable Properties of Polyimine Enhanced by Biomimetic Modification of Graphene Oxide Sheets/Silicon Carbide Nano-Whiskers

**DOI:** 10.3390/nano12244486

**Published:** 2022-12-18

**Authors:** Si Zhang, Shiyu Ji, Zifan Wang, Jian Zhang, Wei Zhao, Chaoshuai He, Yun Chen

**Affiliations:** School of Mechanical Engineering, Jiangsu University of Science and Technology, Zhenjiang 212000, China

**Keywords:** mechanical properties, recyclability, biomimetic, polyimine, graphene oxide

## Abstract

Inspired by the mineral bridge between hard phase layers of natural nacre, the biomimetic modified silicon carbide nano-whiskers (MSiCw)/graphene oxide sheets (MGO) reinforced polyimine (PI) composites (MSiCw-MGO-PI) were successfully prepared by heat-pressing at room temperature, which confirmed by FTIR, XPS, and XRD tests. According to the results of mechanical tests, the composites with filling weights of MSiCw and MGO, which were found to be 1% and 0.3%, presented tensile strength of 94.27 MPa, which was 32% higher than the matrix. With the additional weights amount of 1%MSiCw and 0.2%MGO, the impact strength of the composites reached 17.46 KJ/m^2^, which was increased by 81% compared with the matrix. In addition, the reinforcing mechanisms, such as the bridging principle and mechanism of whiskers pulling out, were investigated by analyzing the fracture surface of MSiCw-MGO-PI composites. The results showed that MSiCw and MGO can synergistically improve the mechanical properties of the composites. In addition, the recyclability of the composites valued by the mechanical properties of the composites from regrinding and heat pressing showed that three generations of MSiCw-MGO-PI composites can still maintain high mechanical properties on account of the better dispersion of the reinforcing phases in the matrix from regrinding.

## 1. Introduction

Lightweight, high performance and high environmental protection composites are the research emphasis in the fields such as astronautics and national defense [1]. Biomimetic materials, as emerging composites, are expected to be typical representatives of lightweight and high-strength materials on account of their hierarchical structures at different dimensions. For example, filled gels, including ferrogels, are well-studied materials with biomimetic properties appropriate for magnetic sensor applications and regenerative medicine, where mechanical properties are essential. It is also an important point for the development of substrates for cell cultivation and drug delivery [2,3]. Good magnetic properties of filler can both improve mechanical and magnetic properties of materials [4]. Natural nacre is a naturally occurring material that is mainly formed by the accumulation of calcium carbonate and organic proteins according to the structure of “brick and mud” [5,6]. The stacked microstructure forms an organic–inorganic–organic layered distribution structure [7,8], which is an essential reason for the excellent mechanical properties of nacre [9]. In fact, the excellent mechanical properties of nacres are relatively close not only to their special hierarchical structure but also to the mineral bridges between nature nacres [10]. Mineral bridges through the soft material layers, enabling hard and soft matters to connect, which transfers matter as the nacre grows and plays a vital role in bearing loads at the same time [11]. The internal structure of the organism became a source of inspiration for the design of high-performance composites, so more and more researchers began to mix nanofillers into the material matrix as reinforcing phases to enhance the mechanical properties of composites [12]. Yunhong Liang et al. produced a bionic eagle feather composite material with robust mechanical properties by mixing carbon fiber with epoxy resin [13]. Wang et al. found that adding 40% e-glass fiber to a 60% epoxy resin base can greatly improve the maximum tensile strength and compressive strength of epoxy resin composites [14]. Zhang et al. found that zirconia nanoparticles can increase the toughness of polyimine by 85% [15].

Polyimine (PI) is an emerging thermosetting material, mainly prepared by the polycondensation reaction of aldehydes and amines [16,17,18]. The mechanical properties of polyimine in hardness, tensile strength, impact strength and other mechanical properties are similar to common thermoplastic materials. It is worth noting that polyimine is easy to synthesize and can be shaped by heat pressing under relatively low pressure and temperature. Compared with thermosetting materials, the reversible properties of imines in polyimine composites have plasticity, high ductility, recyclability and self-healing properties [19,20], which meet a wide application in plenty of hot research areas.

Among lots of nano-filler reinforcing phases, graphene sheets (GNPS), as a naturally occurring carbonaceous material with a single-layer two-dimensional honeycomb crystal structure [21], is also the thinnest and strongest material in the world with excellent mechanical properties. Silicon carbide nano-whiskers (SiCw), which are grown by silicon carbide particles under the action of catalysts [22,23], are a kind of widely used nanomaterials. Compared with granular fillers, nano-whisker fillers have not only obvious advantages in magnetic properties [24,25] but also have much higher strength and toughness than granular fillers. Compared with the addition of a single nanoparticle reinforcing phase, adding the above two mixed nanofillers to the matrix is able to enhance the mechanical properties of the composites synergistically. Recent findings indicated that graphene oxide nano-sheets (GO) can serve as excellent material for the assembly of SiC particles [26], which effectively exhibited excellent mechanical properties [27,28]. Therefore, adding the modified GO and SiCw to the PI matrix according to the bionic idea from natural nacre is an effective means to improve the mechanical properties of PI, which increases the application field of the polymer [29].

In this paper, based on the bridging principle between soft and hard materials and the mechanism of fiber pulling out of natural nacres, MGO and MSiCw were added to polyimine by heat pressing to enhance the mechanical properties of the composites. Through the mechanical properties, SEM, and EDX tests of the biomimetic MSiCw/MGO reinforced polyimine (MSiCw-MGO-PI) composites. It can be seen that the additional weights amount of MSiCw and MGO can significantly improve the mechanical properties of the composites. Compared with the existing single-filler reinforcement literature [13], the reinforcement fillers in this work had a good synergistic reinforcement effect. In addition, the composites also have good recyclability, and the test results showed that the composites not only can maintain mechanical strength but also improve elongation at break. This greatly improves the possibility of application of the composites in various fields and fills the gap of insufficient strength of recyclable materials in the current research [30].

## 2. Experimental

### 2.1. Materials

All the chemicals, such as terephthalaldehyde (TA, AR), diethylenetriamine (DETA, AR), and triethylenetetramine (TETA, AR), were purchased from Shanghai Aladdin Biochemical Technology Co., Ltd. (Shanghai, China). Nanographene sheets (GNPs, 99.5%) and silicon carbide nano-whiskers (SiCw, 99.5%) were purchased from Shanghai Coconut Environmental Protection Technology Co., Ltd. (Shanghai, China). 3-Aminopropyltrimethoxysilane (APTMS, AR), concentrated sulfuric acid (H_2_SO_4_, AR), sodium nitrate (NaNO_3_, AR), potassium permanganate (KMNO_4_, AR), hydrochloric acid (HCl, AR), hydrogen peroxide (H_2_O_2_, AR), acetone (C_3_H_6_O, AR), and N, N-dimethylformamide (DMF, AR) were purchased from Sinopharm (Shanghai, China). All the reagents were analytically graded and used without further purification [31].

### 2.2. Composites Preparation 

The fabricated method of polyimine (PI) was as follows: Before the experimental procedure, TA powder was carefully cleaned with Anhydrous ethanol for 10 min and was, finally, dried in a vacuum oven at 60 °C. The fabricated method of polyimine was as follows. TA, DETA, and TETA were mixed in 60 °C ethyl acetate in a magnetic stirrer (500 rpm/min) according to the ratio of 3:0.9:1.4 with no catalyst added [32]. After the reaction process, the products were collected using a funnel and washed with deionized water several times. The PI powders were then obtained after drying the products at 60 °C overnight.

Graphene oxide (GO) was prepared by the modified Hummers method [33]. The fabricated method of modified graphene oxide (MGO) was as follows: An amount of 50 mg of GO was added to 50 mL of a deionized aqueous solution, and the mixture was dispersed by ultrasound for 2 h. After dispersion, 2 mL APTMS was added to GO aqueous solution, then the mixed solution was stirred magnetically for 4 h in a magnetic stirrer (500 rpm/min) at room temperature to complete the surface modification. After the surface modification, the samples were washed (medium; water, acetone; time 25 min) until the pH value of the filter solution was neutral. The thoroughly washed solid was filtered off and air-dried overnight in an oven at 60 °C to obtain MGO.

The fabricated method of modified silicon carbide nano-whiskers (MSiCw) was as follows: 28 mL DMF and 4 g SiCw micro-powder were added to the beaker to disperse in ultrasound for 2 h, then APTMS (0.2 mL) was taken into SiCw solution for dispersion at a temperature of 90 °C for 6 h (500 rpm/min). After the end of the reaction, the product was vacuum filtered and dispersed by multiple ultrasonic (ultrasonic medium; water, acetone; time 30 min) and centrifugal washing (medium; water, acetone; time 25 min) until the PH of the filtrate was neutral. The samples were dried in an oven at 105 °C for 12 h to obtain MSiCw.

In the fourth step, the composites can be performed utilizing the following detailed process: in a typical procedure, MSiCw (0.1 g), MGO (30 mg) and PI powder (10 g) were mixed in a beaker with ethanol, then the mixed solution was stirred magnetically for 2 h in a magnetic stirrer (500 rpm/min) under magnetic condition. The mixture was poured into an incubator for drying after stirring, and the uniform powder product can be obtained through an 80-mesh screen. The biomimetic MSiCw/MGO reinforced polyimine composites (MSiCw-1-MGO-0.3-PI) (MSiCw-X-MGO-Y-PI, X: MSiCw content, Y: MGO content) can be obtained by heat pressing molding at 80 °C, 9 MPa. The control composites were prepared using the above method, except the MSiCw or MGO was replaced by SiCw or GO.

### 2.3. Test Methods

The hardness test was performed on the HBRVS-18T7.5 Digital Display Brinell hardness tester. The HRR model was used for the test, together with a ball indenter with a diameter of 12.70 mm.

The tensile strength test was performed on a WDW-005 type microcomputer-controlled electronic universal testing machine (Jinan Hengsi Shanda Instruments Co., Ltd., Jinnan, China), and the effective size of the samples was 35 * 5 * 4 mm^3^. The test speed was 0.02 mm/min.

The Charpy pendulum impact tester was adopted to test impact strength in this experiment, and the pendulum energy was 7.5 J. 

Recycle tests of MSiCw-MGO-PI composites were performed on the equipment, which tested hardness, impact strength and tensile strength. Three generations of test samples were formed from the previous generation of the composites by repolishing and heat pressing.

The Fourier Transform Infrared (FTIR) spectroscopy test was performed on a Fourier infrared spectrometer produced by Thermo Fisher (Waltham, MA, USA). Samples of SiCw, MSiCw, PI and MSiCw-MGO-PI composites were prepared by KBr compression. 

XRD test was performed on the XRD-6000 (Japan). The test conditions were as follows: voltage 40 KV, current 30 mA, anode Cu, wavelength λ = 0.154 nm, scanning angle 10–80° at room temperature, scanning speed 4°/min. Both GO and MGO were in powder form.

The X-ray photoelectron spectroscopy (XPS) test was performed on an X-ray photoelectron spectrometer produced by Thermo Fisher in the United States. Samples preparation of MSiCw-MGO-PI composites powder were carried out by pressing (pressure: 5 MPa, time: 10 s).

The fracture of the PI and MSiCw-MGO-PI composites were observed by scanning electron microscopy (SEM) (XL-30 ESEM FEG, FEI). The elemental mapping was performed with Genesis 2000 (EDAX Corporation, Tokyo, Japan). All samples were coated with gold before scanning electron microscopy.

## 3. Results and Discussion

### 3.1. Preparation of Biomimetic Modified Graphene Oxide/Silicon Carbide Whisker Reinforced Polyimine Composites

**Figure 1:**
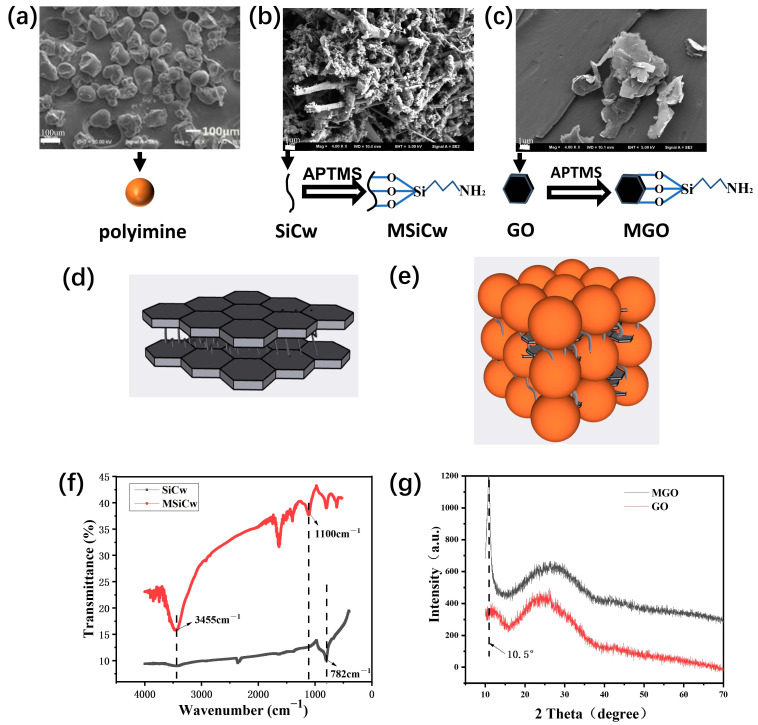
SEM images of polyimine (**a**), SiCw (**b**) and GO (**c**). Microscopic diagram of a natural nacre (**d**). Illustration of interface design of bioinspired layered (**e**). FTIR spectra: SiCw and MSiCw (**f**). XRD spectra: GO and MGO (**g**).

Figure 1a–c shows SEM pictures of PI, SiCw and GO raw materials. As can be seen, the size of PI was on a micron scale, while the size of SiCw and GO were on a nanometer scale. In addition, SiCw and GO were modified by APTMS to obtain better dispersion, thereby better interacting with the matrix. Chen, P.-Y. et al. found that the cross-linking network and layered structure of organic matter from SEM pictures of natural nacre was the main reason for the excellent mechanical properties of natural nacre [34]. In this work, MSiCw and MGO acted as bridges and layers [Figure 1d,e] which mimicked the natural nacre. To demonstrate the successful preparation of the reinforcing phases, MSiCw and SiCw were characterized by FTIR. As shown in Figure 1f, the spectra of MSiCw and SiCw both had vibration peaks caused by the Si-C bond at 782 cm^−1^. However, the infrared spectra of MSiCw showed that the vibration peak caused by the Si-O bond occurred at 1100 cm^−1^ from APTMS, indicating that MSiCw was successfully prepared. XRD test was used to analyze GO and MGO reinforcing phases. As shown in Figure 1g, The spectra of GO exhibited a strong diffraction peak at 2θ = 10.5°, indicating an interlayer spacing of about 0.84 nm based on Bragg’s law. After the functionalization of GO by APTMS, the spectra of MGO showed no obvious peak at 2θ = 10.5°, suggesting GO has exfoliated completely and the functionalized GO has stacked together loosely [35,36]. It was also found that the peak phase of MGO shifted to the right compared with GO [37], which represented the enhancement of the interlayer spacing, indicating that the embedding of amino functional groups in APTMS could open the interlayer spacing of GO to increase the spacing and enhance the dispersion of composites. Therefore, the MGO reinforcing phase was successfully prepared.

**Figure 2:**
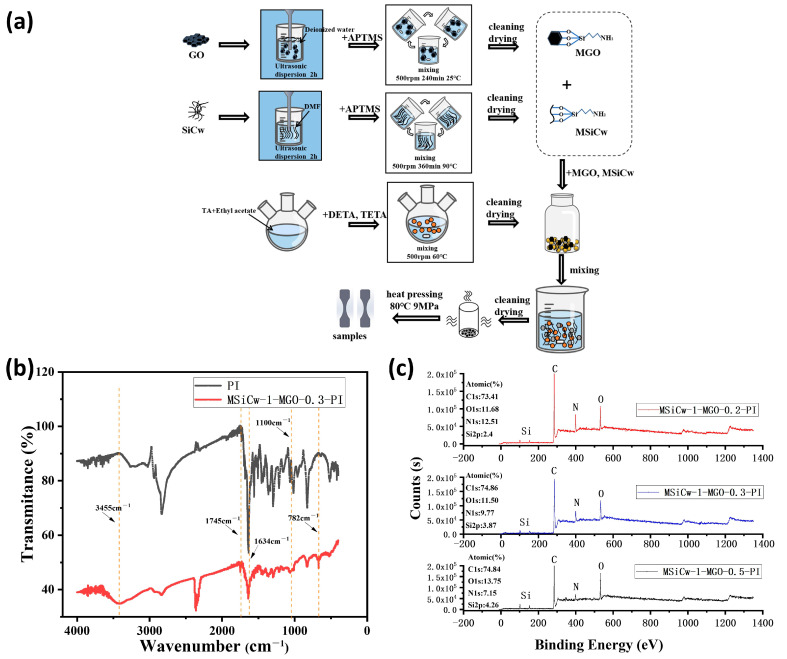
Flow chart of preparation of MSiCw-MGO-PI composites (**a**). FTIR spectra: polyimine and MSiCw-1-MGO-0.3-PI (**b**). Wide-scan survey XPS spectrum of MSiCw-MGO-PI composites (**c**).

The preparation process of MSiCw-MGO-PI composites is shown in Figure 2a. The preparation of PI powder was executed according to the condensation of ammonia and aldehyde at 60 °C. APTMS was added into SiCw and GO solutions for surface modification by stirring and ultrasonic dispersion. In order to verify the successful preparation of the composites, the PI matrix and MSiCw-1-MGO-0.3-PI composites were characterized by FTIR. The infrared test results are shown in Figure 2b. It can be concluded from the infrared curves of PI and MSiCw-1-MGO-0.3-PI that the aldehyde group characteristic peak (1693 cm^−1^) on TA disappeared. At the same time, a vibration peak (C=N) caused by the imine bond occurred at 1634 cm^−1^, which was from the polycondensation reaction of the primary amine groups from DETA and TETA and the aldehyde groups from TA. The above results indicated that PI had been successfully prepared. However, from the infrared peak of MSiCw-1-MGO-0.3-PI, telescopic vibration peaks occurred at 782 cm^−1^ (Si-C bond) and 1100 cm^−1^ (Si-O bond) [38] from MSiCw. Telescopic vibration peaks occurred at 3455 cm^−1^ (C-OH bond) and 1745 cm^−1^ (C=C bond) caused by MGO [39,40,41]. XPS curves of MSiCw-MGO-PI composites are shown in Figure 2c. The distinctive peaks of MSiCw-MGO-PI composites at 285.8 eV, 399.7 eV, 532.5 eV and 102.3 eV confirmed the existence of carbon (C), nitrogen (N), oxygen (O) and Silicon (Si) element. From the element content in Figure 2c, it can be seen that the content of the Si element gradually increased after the continuous addition of MGO. Since the Si-O bond in APTMS successfully reacted with GO, resulting in the Si element in MGO. When the amount of MSiCw was constant, the Si element content in the composites increased with the increase of the amount of MGO, which confirmed that the composites with various MSiCw and MGO additional weights were successfully produced. The texts of XPS and FTIR both indicated that the reinforcing phases had been successfully embedded in the matrix, which improved that MSiCw-MGO-PI composites have been successfully prepared. 

### 3.2. Tensile Strength Analysis

**Figure 3:**
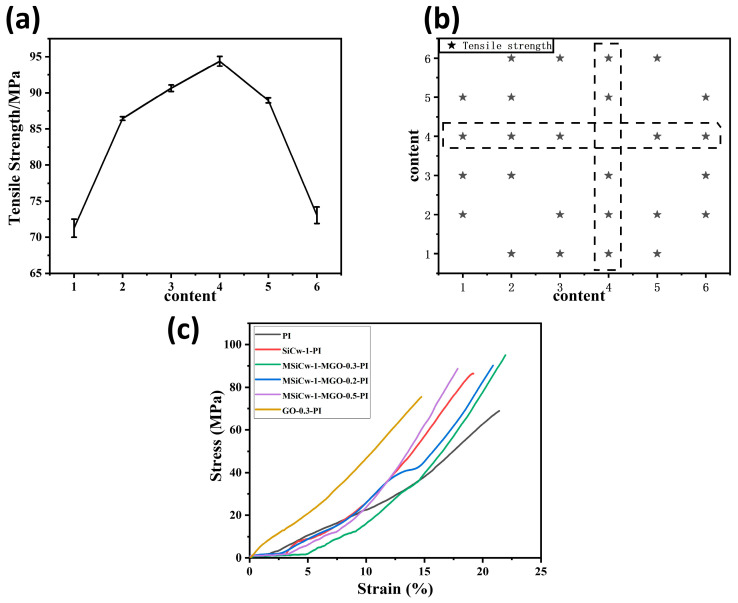
Tensile strength (**a**) and stress–strain (**c**) curves of different types of polyimine composites: Polyimine (1), SiCw-1-PI (2), MSiCw-1-MGO-0.2-PI (3), MSiCw-1-MGO-0.3-PI (4), MSiCw-1-MGO-0.5-PI (5), GO-0.3-PI (6). The statistical significance (*P* < 0.05) for the pairwise comparisons of tensile strength (**b**) (stars) among the composites with different reinforcing phases content.

The mechanical properties of PI and composites with various reinforcing phases content are listed in Table 1. Figure 3a shows the tensile strength of the composites under various additions of reinforcing phases. The tensile strength of the composites increased first and then decreased with the addition of MGO (0.2–0.5%). The stress versus strain curves of the composites with various enhancing phases added is provided in Figure 3c. As observed, the incorporation of MSiCw and MGO had a positive influence on the tensile strength compared to the matrix and the composites with the direct addition of SiCw or GO. When the addition of MSiCw and MGO reached 1% and 0.3%, the tensile strength of the composites ascended to a maximum value (94.27 MPa), which was 32% higher than that of the matrix (71.25 MPa). Most of the pairwise comparisons on tensile strength were significant (*p* < 0.05) among the samples [Figure 3b]. From the above test results, it can be seen that the addition of MSiCw and MGO had an overall improvement effect on the tensile properties of PI.

**Figure 4:**
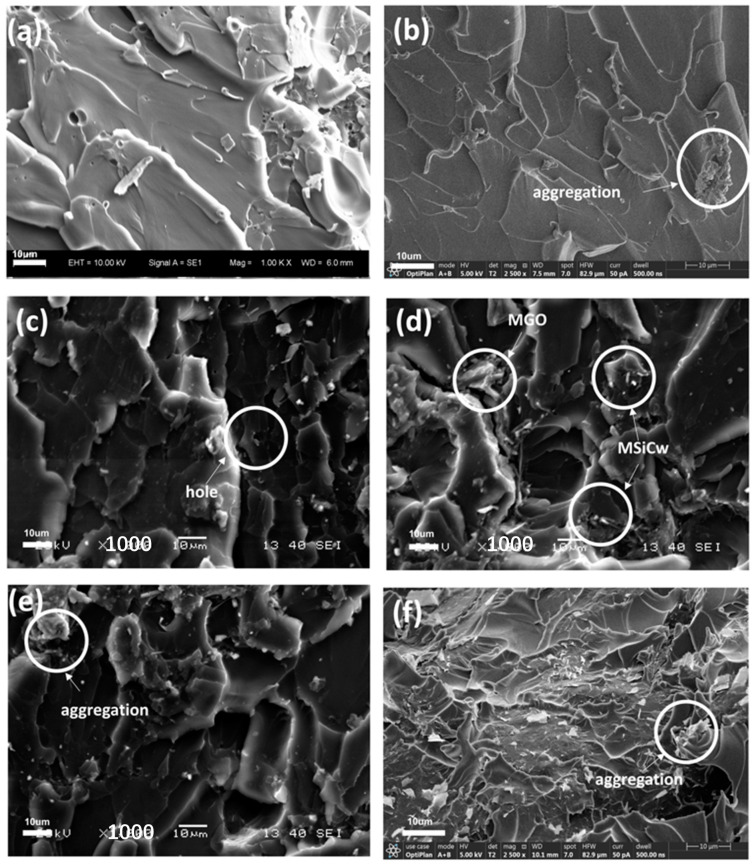
SEM images of tensile fracture surfaces of PI (**a**), SiCw-1-PI (**b**), MSiCw-1-MGO-0.2-PI (**c**), MSiCw-1-MGO-0.3-PI (**d**), MSiCw-1-MGO-0.5-PI (**e**), GO-0.3-PI (**f**).

To investigate the mechanism of the enhancement, tensile fracture surface images of the composites tested by SEM were presented in Figure 4. In general, it can be observed from the apparent river patterns observed in all SEM images that the fracture mode of the matrix and composites were brittle fractures. The fracture surfaces relative to the direct addition of SiCw or GO [Figure 4b,f] after the damage was relatively smooth, indicating weak interfacial interactions between enhancing phases and the matrix. While in the case of MSiCw-MGO-PI composites [Figure 4c–e], it can be observed that there were more obvious folds and cross-linking pores [42], which significantly increased the filling area between the matrix and the reinforcing phases and increased the interfacial binding force [43]. To be specific, modified reinforcing phases were advantageous to withstand external forces and disperse pressure for the matrix, thereby enhancing the tensile strength of the composites as a whole. It can be found from Figure 4d that MGO and MSiCw occurred around the cracks, which effectively prevented crack propagation [44,45,46] and consumed the energy generated during the stretching process according to the whiskers pull-out mechanism [12]. What is more, MSiCw also acted as a bridge to connect the matrix and MGO, which consumed the work conducted by the tensile force during stretching [47]. Therefore, the addition of MGO and MSiCw had a good synergistic enhancement effect on the mechanical properties of the composites. However, it can be found from Figure 4e that MSiCw and MGO would aggregate with excess MGO amount added. The aggregation would reduce the interface interaction between the matrix and the reinforcing phases and result in stress concentration, which would impair the overall mechanical properties of the composites [27,48]. Figure 4e also showed that MSiCw and MGO have different distributions in the matrix, leading MGO and MSiCw to fail to play a synergistic enhancement effect. This explained the reason why the overall tensile strength of the composites decreased slightly when the amount of MGO was added excessively.

### 3.3. Impact Strength and Hardness Analysis

**Figure 5:**
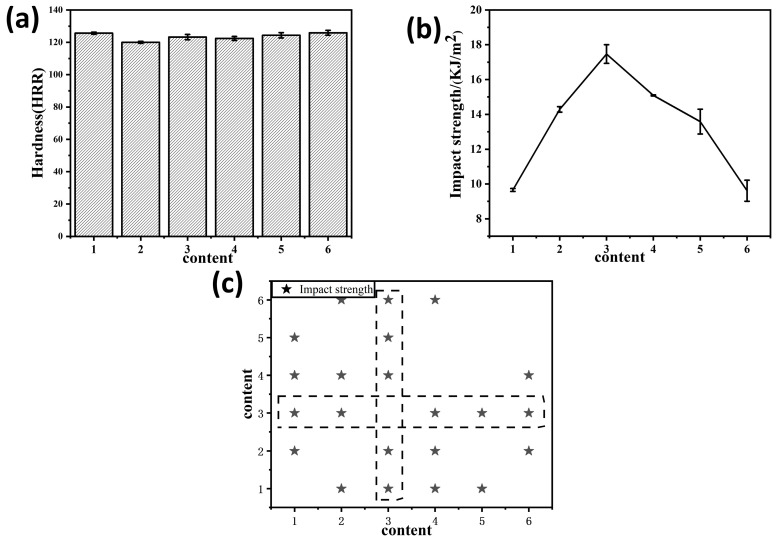
Hardness (**a**) and impact strength curves (**b**) of different types of polyimine composites: polyimine (1), SiCw-1-PI (2), MSiCw-1-MGO-0.2-PI (3), MSiCw-1-MGO-0.3-PI (4), MSiCw-1-MGO-0.5-PI (5), GO-0.3-PI (6). The statistical significance (*P* < 0.05) for the pairwise comparisons of impact strength (**c**) (stars) among the polyimine composites with different reinforcing phases added.

In order to further evaluate the mechanical properties of MSiCw-MGO-PI composites, hardness and impact strength tests were employed. Figure 5a depicts the hardness of composites with different amounts of reinforcing phases added. The hardness of composites was maintained at about 123HRR, which changed slightly with various enhancing phases added. Figure 5b shows the change curve of the impact strength of the composites with various addition of reinforcing phases added. With the addition of MGO, the impact strength of the composites showed a trend of first increasing and then decreasing. When 1% MSiCw and 0.2% MGO were added, the impact strength of the composites reached 17.46 KJ/m^2^, which was 81% higher than that of the matrix (9.65 KJ/m^2^), showing a huge improvement. With the continuous addition of MGO, the impact strength had a downward trend, which indicated that the excess addition of reinforcing phases can no longer enhance the impact strength of the matrix. Pairwise comparison of the test results of impact strength [Figure 5c] under various addition of reinforcing phases showed that most test results were significant (*p* < 0.05).

**Figure 6:**
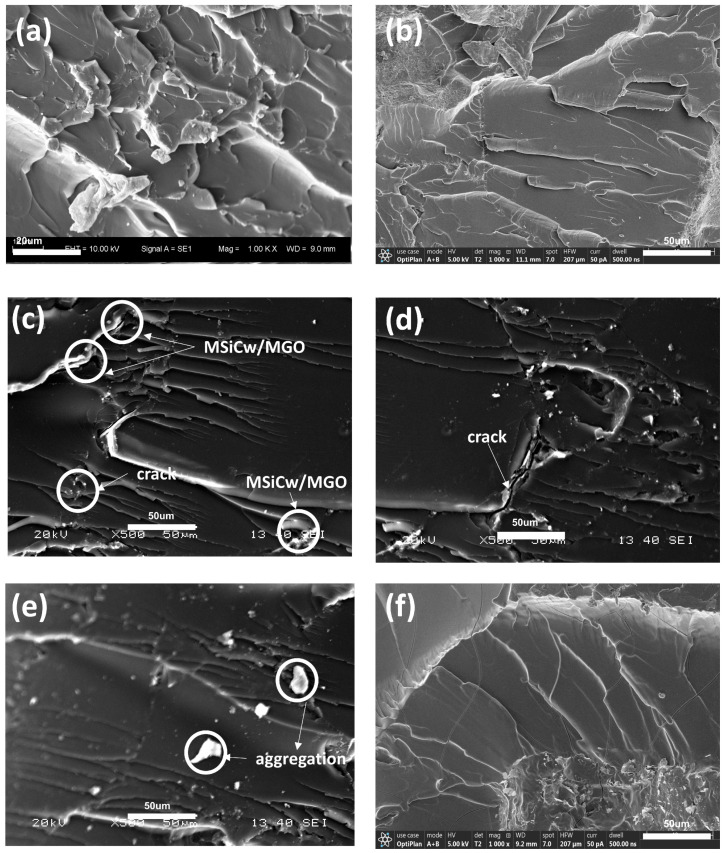
SEM images of impact fracture surfaces of PI (**a**), SiCw-1-PI (**b**), MSiCw-1-MGO-0.2-PI (**c**), MSiCw-1-MGO-0.3-PI (**d**), MSiCw-1-MGO-0.5-PI (**e**), GO-0.3-PI (**f**).

Figure 6a–f depicted SEM images of the impact fracture surfaces of the composites with different reinforcing phases added. Similar to the tensile fracture surfaces, the uniform distribution of MGO and MSiCw increased interfacial binding force on account of the interfacial interactions between the reinforcing phases and the matrix. In addition, MSiCw and MGO were gripped by the matrix before impacting. With the increase of impact load, the crack appeared from the matrix and was halted by MGO [Figure 6c]. During the impact process, the broken MSiCw was pulled out against the frictional grip of the matrix and MGO, which improved the impact strength of the composites. Additionally, the cracks can be pinned at the interfaces of the MSiCw/MGO and PI matrix, leading to extra energy dissipation. These phenomena manifest that crack deflection, crack pining, and whiskers pull-out mechanisms were significant tensile strength mechanisms of MGO/MSiCw. Therefore, when the amount of MSiCw and MGO was 1% and 0.2%, the impact strength of the composites reached the maximum. Consistent with the results of the tensile test, excess MGO would lead to a decrease in impact strength, which was presumably caused by the agglomeration of MGO and MSiCw [Figure 6e]. Not only that, the agglomeration and uneven distribution of MGO and MSiCw decreased synergistic enhancement effects, resulting in the inapparent enhancement effect of the composites.

**Figure 7:**
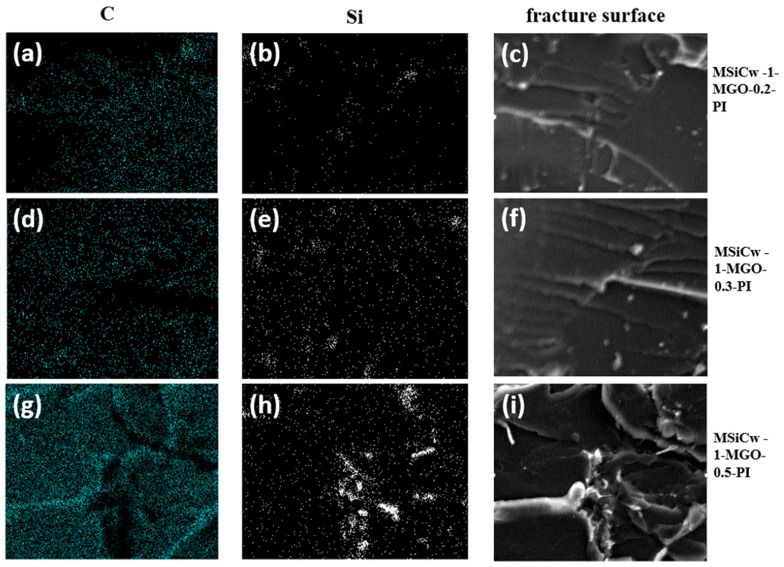
Elemental mappings of C (**a**,**d**,**g**) and Si (**b**,**e**,**h**) of fracture surface MSiCw-1-MGO-0.2-PI (**c**), MSiCw-1-MGO-0.3-PI (**f**) and MSiCw-1-MGO-0.5-PI (**i**).

In order to better explain the influence of the dispersion of various reinforcing phases added in the matrix, elemental mappings of carbon (C) and Silicon (Si) were performed to observe the impact fracture surfaces of the composites. It can be found that with the increase of MGO content, the distribution of Si elements will also be different. For MSiCw-1-MGO-0.5-PI, the elemental mapping of Si [Figure 7h] revealed an agglomerated pattern relative to the generated cracks in Figure 7i. The aggregation of MGO and MSiCw will result in stress concentration, thus affecting mechanical properties.

Together, according to the results of tensile and impact strength tests, the mechanical properties of composites can be greatly enhanced by MSiCw and MGO. The main reason was that adding APTMS can greatly improve the dispersion of the reinforcing phases in the composites. Not only that, MSiCw and MGO can synergistically play the role of bridging and preventing crack propagation [49]. Yet the optimal amount to achieve the best performance for a specific property may vary, depending on the testing conditions, which led to various fracture surfaces as observed by SEM test. For instance, the whisker pull-out mechanism can effectively consume energy, leaving holes behind [Figure 4c]. In contrast, such holes were not evident when cross-sectional forces of impact tests were employed.

### 3.4. Recycle Tests of Mechanical Properties

**Figure 8:**
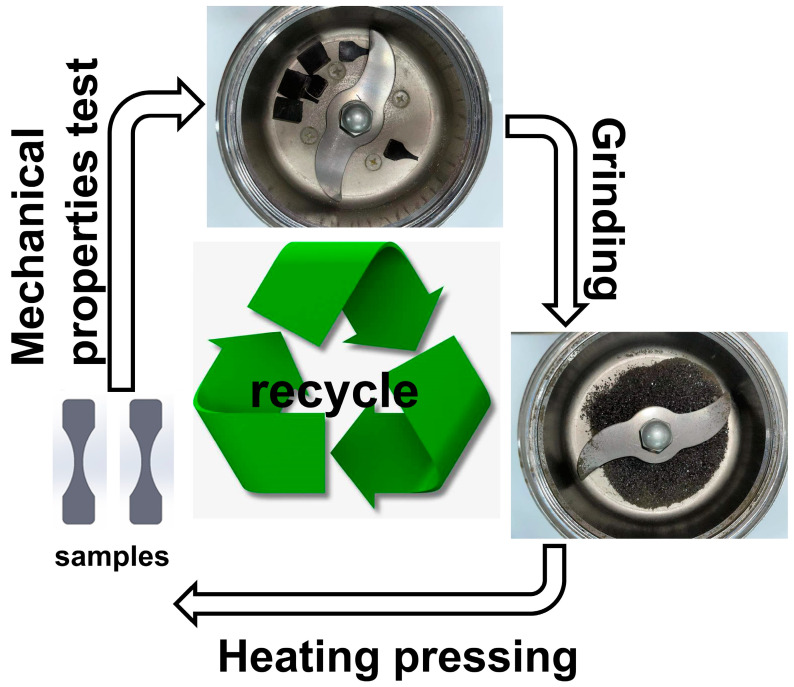
Circulation flow chart of MSiCw and MGO reinforced PI composites.

**Figure 9:**
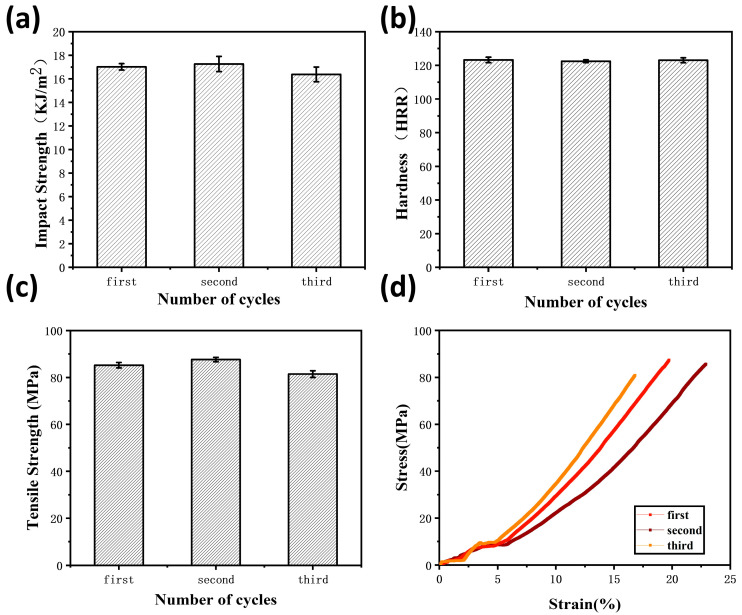
Impact strength (**a**), hardness (**b**), tensile strength (**c**) and stress–strain (**d**) of three generations of MSiCw-1-MGO-0.3-PI composites.

In order to verify the recyclability of the composites, the mechanical properties of the third generation of MSiCw-1-MGO-0.3-PI composites were tested [Figure 8]. Mechanical Properties of MSiCw-1-MGO-0.3-PI composites after three generations are listed in Table 2. The recycled composites could be obtained through repeated grinding and heat pressing of the previous generation of samples according to the thermoplasticity of imine bonds. Generally, the mechanical properties of recyclable composites will decrease during the recycling process. However, as shown in Figure 9a, the impact properties of the second-generation MGO/MSiCw reinforced PI composites showed an upward trend. The impact strength of the third generation was lower than that of the second generation but close to the first generation. Tensile and hardness test results showed that the third-generation cyclic composites can be well maintained at about 80 MPa and 123 HRR [Figure 9b,c], which were not affected obviously by the number of cycles. Combined with the stress–strain curve in Figure 9d, it can be seen that in the process of secondary recycling, the tensile strength and elongation at the break of the composites exceed the first-generation composites. Reasons for the increase of the elongation at the break of the composites were attributed to the decrease of crosslinking density between imine bonds. The recycling process may redistribute the MGO and MSiCw among the matrix, which caused the above changes in the mechanical properties of the composites. The reinforcing phases had better interface interaction and synergistic effect with the matrix during recycling, which well enhanced the binding force between the reinforcing phases and the matrix, thus improving the mechanical properties. From the point of view of impact strength, tensile strength and hardness among three generations of mechanical tests, the mechanical properties of the composites did not decrease significantly in the process of recycling, sometimes the mechanical properties of which were better than the first generation.

## 4. Conclusions

The successful production of MSiCw and MGO resulted in better dispersibility of the microspheres, which increased the filling areas between fillers and PI. The enhancing effect is highly influenced by the distribution of the MSiCw/MGO in the PI matrix according to the bridging principle, mechanism of whiskers pulling out and mechanism of significant enhancement of MSiCw/MGO. The uniform fillers distribution and optimized tensile strength were obtained with filling weights of 1% MSiCw and 0.3% MGO. With the additional amount of 1% MSiCw and 0.2% MGO, the impact strength of the composites reached the maximum, which was increased by 81% compared with the matrix. In addition, incorporated with MSiCw/MGO, the composites retain recyclability after three generations of MSiCw-1-MGO-0.3-PI with high impact and tensile strength. The test results showed that the composites can improve the elongation at the break of the composites without reducing their tensile strength and hardness owing to better dispersion of the reinforcing phases in the matrix from regrinding.

## Figures and Tables

**Table 1:** Mechanical Properties of PI and composites with various reinforcing phases content.

Material	Tensile Strength (MPa)	Impact Strength (KJ/m^2^)	Rockwell Hardness (HRR)
PI	71.25 ± 1.25	9.655 ± 0.085	125.66 ± 0.68
SiCw-1-PI	86.45 ± 0.25	14.2875 ± 0.158	120.01 ± 0.61
MSiCw-1-MGO-0.2-PI	90.55 ± 0.35	17.465 ± 0.535	123.26 ± 1.62
MSiCw-1-MGO-0.3-PI	94.275 ± 0.78	15.09 ± 0.03	122.41 ± 1.24
MSiCw-1-MGO-0.5-PI	88.95 ± 0.35	13.585 ± 0.715	124.35 ± 1.62
GO-0.3-PI	73.05 ± 1.15	9.62 ± 0.605	125.9 ± 1.53

**Table 2:** Mechanical Properties of MSiCw-1-MGO-0.3-PI composites after three generations.

Generation	Tensile Strength (MPa)	Impact Strength (KJ/m^2^)	Rockwell Hardness (HRR)
first	85.25 ± 1.15	17.02 ± 0.27	123.26 ± 1.62
second	87.65 ± 0.95	17.56 ± 0.61	122.46 ± 0.82
third	81.45 ± 1.42	16.38 ± 0.62	123.04 ± 1.48

## Data Availability

The data used to support this study are included within the article.
